# Supplementary material for: Molecular mechanism of bacteriophage contraction structure of an S-layer–penetrating bacteriophage
Source: Life Sci Alliance. 2025 Mar 26;8(6):e202403088. doi: 10.26508/lsa.202403088 (PMC11948020; doi:10.26508/lsa.202403088)
Supplement: Supplementary file 2 [file LSA-2024-03088_TableS1.docx]

**Table S1.** Cryo-EM data collection, refinement and validation statistics.

|  | **Extended Phage**  **Capsid I1**  **(EMD-51191)**  **(PDB 9GAY)** | **Extended Phage**  **Portal C5**  **(EMD-51193)**  **(PDB 9GB0)** | **Extended Phage**  **Portal C12**  **(EMDB-51196)**  **(PDB 9GB3)** | **Extended Phage**  **Neck**  **(EMDB-51200)**  **(PDB 9GB7)** | **Extended Phage**  **Tail**  **(EMD-51194)**  **(PDB 9GB1)** | **Extended Phage**  **Baseplate**  **(EMD-51195)**  **(PDB 9GB2)** | **Extended Phage**  **Needle**  **(EMDB-51138)**  **(PDB 9G8S)** | **Contracted Phage**  **Capsid I1**  **(EMDB-51192)**  **(PDB 9GAZ)** | **Contracted Phage**  **Portal C12**  **(EMDB-51197)**  **(PDB 9GB4)** | **Contracted Phage**  **Neck**  **(EMDB-51198)**  **(PDB 9GB5)** | **Contracted Phage**  **Tail**  **(EMDB-51199)**  **(PDB 9GB6)** |
| --- | --- | --- | --- | --- | --- | --- | --- | --- | --- | --- | --- |
| **Data collection and processing** |  |  |  |  |  |  |  |  |  |  |  |
| Magnification | 81,000 | 81,000 | 81,000 | 81,000 | 81,000 | 81,000 | 81,000 | 81,000 | 81,000 | 81,000 | 81,000 |
| Voltage (kV) | 300 | 300 | 300 | 300 | 300 | 300 | 300 | 300 | 300 | 300 | 300 |
| Electron exposure (e–/Å2) | 42 | 42 | 42 | 42 | 42 | 42 | 42 | 42 | 42 | 42 | 42 |
| Defocus range (μm) | 1.5-3 | 1.5-3 | 1.5-3 | 1.5-3 | 1.5-3 | 1.5-3 | 1.5-3 | 1.5-3 | 1.5-3 | 1.5-3 | 1.5-3 |
| Pixel size (Å) | 1.06 | 1.06 | 1.06 | 1.06 | 1.06 | 1.06 | 1.06 | 1.06 | 1.06 | 1.06 | 1.06 |
| Symmetry imposed | I1 | C5 | C12 | C6 | C6 + helical | C6 | C3 | I1 | C12 | C6 | C6 + helical |
| Initial particle images (no.) | 61,489 | 70,251 | 70,251 | 68,159 | 178,796 | 19,276 | 19,276 | 42,410 | 31,079 | 30,325 | 128,963 |
|  |  |  |  |  |  |  |  |  |  |  |  |
| Final particle images (no.) | 61,489 | 23,343 | 38,968 | 23,724 | 157,557 | 19,276 | 17,759 | 38,654 | 26,404 | 30,325 | 48,919 |
| Map resolution (Å) | 3.74 | 3.23 | 2.64 | 3.40 | 2.71 | 3.43 | 3.96 | 3.28 | 2.88 | 3.27 | 4.11 |
| FSC threshold | 0.143 | 0.143 | 0.143 | 0.143 | 0.143 | 0.143 | 0.143 | 0.143 | 0.143 | 0.143 | 0.143 |
|  |  |  |  |  |  |  |  |  |  |  |  |
| **Refinement** |  |  |  |  |  |  |  |  |  |  |  |
| Initial model used (PDB code) | de novo | de novo | de novo | de novo | de novo | de novo | de novo | de novo | de novo | de novo | de novo |
| Model resolution (Å)  FSC threshold | 4.1  0.5 | 3.5  0.5 | 2.8  0.5 | 3.7  0.5 | 3.0  0.5 | 3.8  0.5 | 3.7  0.5 | 3.5  0.5 | 3.2  0.5 | 3.5  0.5 | 5.0  0.5 |
| Model resolution range (Å) |  |  |  |  |  |  |  |  |  |  |  |
| Map sharpening B factor (Å2) | -160.8 | -71.7 | -114.6 | -86.5 | -77.7 | -100.0 | -103.9 | -146.9 | -94.9 | -81.4 | -178.5 |
| Model composition  Non-hydrogen atoms  Protein residues  Ligands | 45,319  22,687  2877  0 | 72,370  36,250  4,600  0 | 85,752  43,008  5,340  0 | 193,800  97,188  12,252  0 | 167,328  83,592  10,620  0 | 173,430  86,424  10,974  0 | 193,897  96,508  12,255  1 | 45,235  22,687  2,877  0 | 85,788  43,008  5,340  0 | 193,800  97,188  12,252  0 | 130,590  65,214  8,280  0 |
| B factors (Å2)  Protein  Ligand | 116.60  - | 100.85  - | 62.97  - | 139.26  - | 133.50  - | 188.62  - | 107.10  102.68 | 69.30  - | 111.0  - | 166.0  - | 308.72  - |
| R.m.s. deviations  Bond lengths (Å)  Bond angles (°) | 0.004  0.637 | 0.004  0.640 | 0.00  0.00 | 0.003  0.615 | 0.006  0.605 | 0.004  0.593 | 0.003  0.581 | 0.004  0.664 | 0.004  0.622 | 0.004  0.636 | 0.004  0.652 |
| Validation  MolProbity score  Clashscore  Poor rotamers (%) | 1.47  3.71  0.00 | 1.56  3.96  0.00 | 1.07  2.78  0.00 | 1.35  4.427  0.00 | 1.50  4.94  0.00 | 1.53  4.42  0.00 | 1.28  2.67  0.00 | 1.37  2.47  0.00 | 0.89  1.52  0.00 | 1.46  4.59  0.01 | 1.52  2.58  0.00 |
| Ramachandran plot  Favoured (%)  Allowed (%)  Disallowed (%) | 95.50  4.43  0.07 | 94.55  5.45  0.00 | 98.47  1.53  0.00 | 97.23  2.77  0.00 | 96.42  3.58  0.00 | 95.58  4.42  0.00 | 96.49  3.45  0.00 | 95.11  4.71  0.18 | 98.19  1.81  0.00 | 96.51  3.49  0.00 | 95.63  4.26  0.11 |
| CC map-to-model  (volume) | 0.74 | 0.83 | 0.81 | 0.78 | 0.75 | 0.84 | 0.76 | 0.81 | 0.77 | 0.75 | 0.83 |
