# Supplementary material for: Molecular mechanism of bacteriophage contraction structure of an S-layer–penetrating bacteriophage
Source: Life Sci Alliance. 2025 Mar 26;8(6):e202403088. doi: 10.26508/lsa.202403088 (PMC11948020; doi:10.26508/lsa.202403088)
Supplement: Supplementary file 3 [file LSA-2024-03088_Supplemental_Data_2.docx]

Supplementary Text

Structure of the capsid and portal

The capsid from extended phages ([Fig 2A](#fig2)) was reconstructed with icosahedral symmetry imposed, to 3.4 Å resolution; it consists of two proteins, the major capsid protein gp49 ([Fig S2A](#figS2)), and the capsid decoration protein gp48 ([Fig S2B](#figS2)). The major capsid protein assembles to form hexameric faces and pentameric vertices in a T = 7 *laevo* icosahedral arrangement ([Fig S2D](#figS2)) ([Caspar & Klug, 1962](#bib_caspar_and_klug_1962)). Thus, the icosahedral asymmetric unit consists of 7 gp49 polypeptides and 7 gp48 polypeptides ([Fig S2C](#figS2)); 6 gp49 subunits are arranged around a quasi-sixfold symmetric axis with the seventh arranged with four additional partners around an icosahedral fivefold symmetry axis. The gp48 polypeptides associate into trimers; two trimers assembled around a quasi-symmetric threefold axis and one polypeptide assembled with partners around a true icosahedral threefold symmetry axis ([Fig S2A](#figS2)). The major capsid protein gp49 adopts the typical HK97 fold ([Suhanovsky & Teschke, 2015](#bib_suhanovsky_and_teschke_2015)), consisting of an axial domain, a peripheral domain, an extended loop, and an N-terminal arm ([Fig S2A](#figS2)). The extended loops point towards the center of the hexameric face ([Fig S2C](#figS2)). In addition, a small insertion domain formed from residues 209–231 ([Fig S2A](#figS2)) forms a β-hairpin that associates with βB of a neighboring capsid decoration protein, contributing to capsid stability.

The capsid decoration protein gp48 forms trimeric protrusions from the capsid surface ([Figs 2](#fig2) and [S2C and D](#figS2)). The decoration protein has structural homology to siphovirus TW1 and podovirus φ29 capsid decoration proteins ([Wang et al, 2018](#bib_wang_et_al_2018); [Xu et al, 2019](#bib_xu_et_al_2019)) ([Table S2](#tblS2)), but lacks the long extension present at the C-terminus of the φ29 protein. Each gp48 trimer interacts intimately with nine neighboring gp49 major capsid subunits ([Figs 2](#fig2) and [S2E](#figS2)). The N-terminal arm of each gp48 decoration protein sits within a hole formed between the axial domain and extended loop of two neighboring gp49 proteins ([Fig S2F](#figS2)). The flexibility of the N-terminal arm is able to accommodate conformational differences in capsid hexameric faces and pentameric vertices.

The portal adopts a unique position within the head and breaks the perfect icosahedral capsid symmetry ([Figs 2B](#fig2) and [S2J](#figS2)). Experimentally, it was located within the capsid by 3D classification (see methods) and was reconstructed to 2.6 Å resolution with C12 symmetry imposed ([Fig 2B and C](#fig2)). Each gp45 portal protein monomer adopts the typical bacteriophage portal fold ([Lebedev et al, 2007](#bib_lebedev_et_al_2007)), consisting of wing (residues 38–215), clip (residues 253–309), stem (residues 228–253 and 310–331), crown (residues 400–469), and channel valve domains (residues 332–366) ([Figs 1C and 2C](#fig1) and [S2G](#figS2)). The portal complex has a minimum inner aperture diameter of 28 Å, which is sufficient to allow DNA to pass through without a conformational change in the portal protein ([Fig S2H](#figS2)).

There is a symmetry mismatch where the dodecameric portal interfaces with the surrounding pentameric capsid vertex ([Fig S2J](#figS2)). The asymmetric reconstruction of the portal shows that the gp48 capsid decoration protein trimers surrounding the portal are still complete trimers, although density for the 15 residue N-terminal tail is not resolved ([Fig S2K](#figS2)). This is reminiscent of the arrangement in Pam3 ([Yang et al, 2023](#bib_yang_et_al_2023)). The portal interacts with the capsid via charge–charge interactions between flexible loops made up of residues 164–172 within the gp49 capsomer and 190–193 in the wing motif of the gp45 portal. Interactions are also formed between the stem of the gp45 portal and the gp48 capsid decoration protein.

2D classes of the capsid show layers of density predicted to be DNA for the extended phage capsid, and no layers for the contracted phage capsid. Asymmetric reconstructions also highlight the presence of density proximal to the portal in the full capsid, which we hypothesise to be the DNA. However, we are unable to resolve grooves in the DNA, and so cannot confidently assign the density. In the emptied capsid and portal reconstructions, this density is missing, suggesting the DNA is fully ejected.

Structure of neck reveals a novel portal interacting protein, as well as some similarity to diverse CISs.

The first complex in the neck is the head-to-tail adaptor gp50, which acts to resolve a near-perfect 12-fold to 6-fold symmetry mismatch between the portal and tail ([Figs 1 and 2B](#fig1) and [S3A](#figS3)). It is formed of an ɑ-helical bundle, a short β-hairpin, and a C-terminal extension of 11 residues ([Fig S3A](#figS3)). The C-terminal extension of gp50 binds directly to the portal, and is tightly pinched between two portal clip domains, forming an extended 5-stranded β-sheet ([Figs 2C](#fig2) and [S3B and C](#figS3)). Overall, the interaction between each gp50 head-to-tail adaptor C-terminal extension and the three portal chains has a total buried surface of ~1,200 Å^2^, ensuring that the capsid remains attached to the neck. The β-hairpin of gp50 (residues 62 to 71) forms a β-barrel in the dodecameric assembly, and accommodates a decrease in symmetry from C12 to C6 between the gp50 and gp51 ([Fig S3E and F](#figS3)). The outside surface of the barrel contains two tyrosine residues per gp50 monomer (Tyr63 and Tyr70), which face the inner lining of the gp51 neck valve hexameric ring. The interacting surface of gp51 is lined with hydrophobic residues that interact with the tyrosines to form a hydrophobic interface ([Fig S3E and F](#figS3)). The interface also contains a salt bridge between Glu68 from two neighboring head-to-tail adaptor (gp50) chains and arginine residues from each neck valve (gp51) chain, Arg7, and Arg48 ([Fig S3F](#figS3)). Gp50 has a similar beta-hairpin to other phage connector proteins, but connects to the portal in a novel way, inserting a beta strand into the portal clip and having a loop linker, such that holes are present in the neck. Despite a similar secondary structure to other connector proteins, Foldseek and DALI structural homology searches find no structural homology between gp50 and other known phage neck protein structures highlighting this connecting protein as being somewhat divergent from other examples ([Table S2](#tblS2)).

The neck valve protein gp51 forms a hexameric complex and forms the narrowest constriction in the neck lumen, ~23 Å in diameter ([Fig S3D and E](#figS3)). gp51 has structural homology to the stopper protein of the *Rhodobacter capsulatus* gene transfer agent complex ([Bardy et al, 2020](#bib_bardy_et_al_2020)), and is made up of an N-terminal helix followed by a β-sheet which bends to form a saddle so that each gp51 monomer sits over three gp53 tail terminator monomers ([Fig S3D](#figS3)).

The tail terminator gp53 forms a hexameric ring linking the neck to the tail ([Figs 2C](#fig2) and [S3G and H](#figS3)), and has two tandem domains. Each domain has a similar fold (RMSD 3.2 Å), with an additional C-terminal extension in the tail-proximal domain II. The C-terminus contains a linker and a β-strand, which inserts into the first layer of the tail sheath ([Fig S3H and I](#figS3)). The linker between domain II and the C-terminal β-strand is formed of 4 residues ([Fig S3C](#figS3)), compared with the 10–13 seen in recently determined pyocin structures ([Ge et al, 2020](#bib_ge_et_al_2020)), AFP ([Desfosses et al, 2019](#bib_desfosses_et_al_2019)), and PVC ([Jiang et al, 2019](#bib_jiang_et_al_2019)) complexes. The β-strand sequence is conserved between the tail terminator and the tail sheath protein gp55, suggesting that the sequence is important for forming the extended interaction between this region with the sheath domain I β-sheet ([Fig S3J](#figS3)).

In order to determine if any large conformational changes are required in the neck proteins to release DNA, the structures in the extended and contracted form were compared ([Fig 1](#fig1)). The overall RMSD between all proteins of the neck in the extended state compared to the contracted state is 0.34 Å, indicating that no large conformational change occurs in this region during phage contraction.

The structure and helical arrangement of the tail subunits is similar to that of typical CISs in the extended state.

The main tail tube protein, gp56, consists of a 7-strand β-sandwich with the conserved fold seen in other Caudovirales tail tubes ([Arnaud et al, 2017](#bib_arnaud_et_al_2017); [Zheng et al, 2017b](#bib_zheng_et_al_2017b); [Kizziah et al, 2020](#bib_kizziah_et_al_2020); [Zinke et al, 2020](#bib_zinke_et_al_2020)) ([Figs 1](#fig1) and [S4A and B](#figS4)). A loop formed of residues 39 to 59 connects each tail tube ring to the ring below, and a short N-terminal helix of one subunit sandwiches each loop of the subunit above, from the outside ([Fig S4A and B](#figS4)). The loop of each tail tube protein interacts with the two neighboring proteins in the same ring plane, and three proteins in the next ring down, forming extensive interactions as seen in other tailed phages ([Fig S4B and C](#figS4)). However, the gp56 tail tube protein lacks both the ɑ-loop and N-loop seen in other myovirus tail tube proteins, as well as the C-terminal arm seen in siphovirus tail tube proteins ([Zinke et al, 2020](#bib_zinke_et_al_2020)) ([Fig S4D](#figS4)). This results in a more open packing between rings of the tail tube ([Fig S4C](#figS4)). This is also reflected in the PISA analysis of buried surface between tail tube monomers, with a reduced buried surface of ~1,900 Å^2^ between rings, compared with ~2,600 Å^2^ for AFP, ~2,500 Å^2^ for pyocin, and ~3,000 Å^2^ for T4 ([Aksyuk et al, 2009](#bib_aksyuk_et_al_2009); [Ge et al, 2020](#bib_ge_et_al_2020); [Weiss et al, 2022](#bib_weiss_et_al_2022)).

The gp55 tail sheath protein is made up of three domains ([Fig 3C](#fig3)). The tail tube proximal C-terminal domain I contains 2 α-helices and a β-sheet. The β-sheet is augmented by N-terminal and C-terminal β-strand insertions from neighboring sheath proteins, forming an interwoven mesh network ([Fig 3B](#fig3)) ([Ge et al, 2015](#bib_ge_et_al_2015)). Domain I also contains a loop (“X-loop”) consisting of residues 368 to 378 (magenta), which is longer in φCD508 than in the sheath domains of other CISs studied (6 residues and 3 residues respectively) ([Fig S4E](#figS4)). This loop pinches the linker of the N-terminal β-strand insertion of a neighboring sheath protein, reducing the possible range of motion of the gp55 sheath subunits. Domain II contains a β-sheet surrounded by short helices, and forms interactions with neighboring sheath domains I and II through charge interactions. Domain III is made up of a β-sandwich with an α-helix insertion and appears less ordered than domain I and II in the extended tail reconstructions as evidenced by the decrease in resolution of this region ([Fig S5A and B](#figS5)). However, domain III could be fitted from the Rosetta predicted structure placed in the density for the first sheath ring next to the baseplate, in which stabilizing interactions form between the sheath distal domain III and the baseplate ([Fig S5C](#figS5)).

φCD508 contains a minimal phage baseplate.

The baseplate of φCD508 is the most complex part of the phage ([Fig 2E](#fig2)). In the extended tail conformation, the baseplate forms a hexagonal assembly with a hub complex of tail initiator proteins (gp61, gp64) ([Fig S7A](#figS7)) towards the center, a wedge complex (gp65a, gp65b, and gp66) ([Fig S7C](#figS7)) and peripheral baseplate components (gp67 and gp68, not modeled) attached radially towards the outside, with a disk-like side profile ([Fig S7C](#figS7)).

The baseplate hub complex acts to bind the tail tube and sheath to the baseplate, and the organization of baseplate hub proteins is conserved between φCD508 and other CISs ([Taylor et al, 2016](#bib_taylor_et_al_2016); [Desfosses et al, 2019](#bib_desfosses_et_al_2019); [Jiang et al, 2019](#bib_jiang_et_al_2019); [Ge et al, 2020](#bib_ge_et_al_2020)). One component of the complex is the tail tube initiator protein gp61. This protein binds to the subunits in the first layer of the tail tube (gp51), with which it shares a similar β-sandwich fold ([Fig S7A](#figS7)). At the C-terminal end of the β-sandwich fold in gp61, a loop (residues 138–165) links to a LysM domain (residues 165–222; [Fig S7A](#figS7)). The fusion of the LysM domain with the tail tube initiator is similar to that of AFP and PVC ([Desfosses et al, 2019](#bib_desfosses_et_al_2019); [Jiang et al, 2019](#bib_jiang_et_al_2019)), but different to T4 in which the LysM domain and tail tube initiator are separate proteins.

A second protein in the baseplate hub complex is the sheath initiator protein gp64. This binds to the terminal ring of sheath proteins and has a similar fold to the sheath protein (gp56) domain I ([Fig S7A](#figS7)). A β-sheet made up of 2 β-strands from gp64, binds β-strands donated from two nearby terminal sheath proteins, gp56 ([Fig S7A](#figS7)). The C-terminal tail forms contacts with the gp61 tail tube initiator’s loop between its β-sandwich domain and lysM domain, stabilizing the tail tube and sheath initiation complex ([Fig S7A](#figS7)).

The baseplate hub complex also provides a platform onto which the wedge is assembled ([Figs 2E](#fig2) and [S7C](#figS7)). The wedge consists of three proteins, two gp65 molecules called triplex 1a and triplex 1b, and one gp66 molecule called triplex 2 ([Fig S7C and D](#figS7)). The wedge proteins are involved in triggering the contraction of the baseplate, and in the tail-extended state bind to each other component of the baseplate, as well as the needle and tail. Each protein comes together via a core bundle (residues 1–63 and 1–47 for gp65 and gp66 respectively) to form the triplex assembly. The core bundle binds to the hub complex, and is made up of a triple stranded parallel ɑ-helical coiled-coil with a typical core of leucine and isoleucine residues ([Harbury et al, 1993](#bib_harbury_et_al_1993)).

gp65 has a “wing” domain (residues 80–184), absent in gp66 ([Fig S7E](#figS7)). The wing domain is structurally related to the T4 gp6 wing domain ([Taylor et al, 2016](#bib_taylor_et_al_2016)), and in the tail-extended state, the respective gp65a and gp65b wing domains adopt a different conformation to bind the tail and needle respectively ([Fig S7D](#figS7)). In order to bind two different proteins, flexible loops in the wing domains can alter their conformation ([Fig S7E](#figS7)). In triplex 1a, the wing contacts the distal domain III of the first sheath protein in the tail. In triplex 1b, alternating wing domains bind to two different sites on the needle hub and tip, breaking the C6 symmetry of the baseplate and resolving with the C3 symmetry of the needle.

Both gp65 and gp66 have trifurcation units (residues 64–79 and 185–281, and 47–156 for gp65 and gp66 respectively). These units form a splayed three-pointed star at the end of the core bundle, allowing each protein’s C-terminus to point non-symmetrically in different directions ([Fig S7D](#figS7)). The C-termini of the two gp65 monomers form dimerisation domains, and six pairs of gp65 dimerisation subunits assemble to form an iris-like circular complex ([Fig S7C](#figS7)). The C-terminus of gp66 is predicted to bind the tail fiber gp67 ([Taylor et al, 2016](#bib_taylor_et_al_2016)), although this interaction is not sufficiently resolved in our reconstructions for model building ([Fig S7F](#figS7)).

The φCD508 needle complex lacks enzymatic domains, and the typical β-helix observed in other CIS needles.

The phage needle is formed of two proteins, a positively charged needle protein (gp62), and a pointed needle tip (gp63) ([Figs 2E](#fig2), [S7B](#figS7), and [S10](#figS10)). The needle protein gp62 functions to resolve the symmetry mismatch between the C6 tail and the C3 needle, forming a hollow trimeric collar around the helical bundle domain of the tip protein ([Fig S7B](#figS7)).

The structure of needle protein gp62 is related to a number of other needle proteins, including gp27 of T4 phage ([Taylor et al, 2016](#bib_taylor_et_al_2016)) ([Table S2](#tblS2)). Each gp62 hub protein forms 2 β-sandwiches, providing six pseudo-symmetric domains to bind to the sixfold symmetric tail tube initiator complex ([Figs S7B](#figS7) and [S10](#figS10)). Each β-sandwich also contains a lobe (residues 89 to 179 and 196 to 261) which forms a collar surrounding the needle tip ([Figs S7B](#figS7) and [S10B](#figS10)). gp62 lacks any predicted enzymatic domains such as the lysozyme or peptidoglycan hydrolase domains observed in other CISs ([Arisaka et al, 2003](#bib_arisaka_et_al_2003); [Guerrero-Ferreira et al, 2019](#bib_guerrero_ferreira_et_al_2019)).

The trimeric needle tip protein gp63 ([Figs S7B](#figS7) and [S10A](#figS10)) adopts a VGR-like fold ([Pukatzki et al, 2007](#bib_pukatzki_et_al_2007)) that differs from that in other phages ([Browning et al, 2012](#bib_browning_et_al_2012)); it consists of an N-terminal helix (residues 6–25), an oligonucleotide/oligosaccharide binding (OB-fold) domain (residues 28–55 and 96–114), and an apex domain (residues 56–95). gp63 lacks the β-helix present in other CIS needles ([Browning et al, 2012](#bib_browning_et_al_2012); [Taylor et al, 2016](#bib_taylor_et_al_2016); [Desfosses et al, 2019](#bib_desfosses_et_al_2019); [Jiang et al, 2019](#bib_jiang_et_al_2019); [Ge et al, 2020](#bib_ge_et_al_2020)), and instead the OB-fold attaches directly to the apex domain ([Figs S7B](#figS7) and [S9A](#figS9)). The apex domain is formed of a short β-hairpin made up of 18 residues, with a HxH motif pointing inwards (His78 and His80 from each chain) ([Figs S7B](#figS7) and [S10A](#figS10)). This HxH motif is also found across many CIS needles, and has been shown to bind an iron in other needle tips with similar arrangements of His pairs ([Browning et al, 2012](#bib_browning_et_al_2012)). A strong density feature is also present between His78 and His80 residues in gp63, that are predicted to represent a metal binding site with a coordinated ion which we have modeled as an iron ion ([Fig S10A](#figS10)).

Tape measure protein

The N-terminus of gp63 forms a 19-residue helix which resides within the tail tube ([Figs S7B](#figS7) and [S10B](#figS10)), forming an internal trimeric helical bundle. Augmenting this bundle are three helices at the C-terminus of the tape measure protein, gp59. There is clear interpretable density for 23 residues within the lumen of the tail tube, the remainder of the tape measure protein appearing disordered. Thus, the six bundled helices form a link between the needle tip and the tape measure protein, with the needle tip acting as a plug prior to contraction and genome release ([Fig S10](#figS10)). The C-terminus of the tape measure protein is predicted to form a number of short β-strands followed by an α-helical “lazo” domain, which could be modeled in the C3 baseplate/needle reconstruction ([Fig S10B and C](#figS10)).

Tail contraction

The contraction ratio of φCD508 bound to receptor is much reduced compared to other CISs. In order to confirm that reduced contraction of φCD508 in the presence of urea represents the fully contracted state, phage were spontaneously contracted in solution by storing particles for extended periods at 4°C, thermally contracted by heating to 70°C, and naturally contracted by binding to fragments of S-layer ([Fig 5](#fig5)). In all cases, TEM showed that phage contracted by 20%.

To determine how φCD508 contracts when binding to the phage’s natural host receptor, the S-layer ([Royer et al, 2023](#bib_royer_et_al_2023)), tomograms were collected from samples frozen 40 min (n = 25) after mixing phage with S-layer fragments ([Fig 5](#fig5)). The lengths of extended and contracted phage tails were measured, and the averages were in agreement with the high resolution structures of φCD508. The extended and contracted tail lengths were approximately 217 nm, and 180 nm respectively ([Fig 1](#fig1)).

The presence or absence of DNA in the phage capsid was also determined. After 5 min, 36% of capsids were full (n = 239), ~5% were partially empty (n = 34), and ~59% were empty (n = 395), whereas after 40 min ~9% were full (n = 49), ~3% were partially empty (n = 17), and ~88% were empty (n = 481). These results demonstrate that phage contraction is not immediately followed by genome release and demonstrate that there are additional step(s) between contraction of the sheath and release of DNA from the capsid.
