# Supplementary material for: Molecular mechanism of bacteriophage contraction structure of an S-layer–penetrating bacteriophage
Source: Life Sci Alliance. 2025 Mar 26;8(6):e202403088. doi: 10.26508/lsa.202403088 (PMC11948020; doi:10.26508/lsa.202403088)
Supplement: Supplementary file 4 [file LSA-2024-03088_TableS2.docx]

**Table S2.** φCD508 proteins structural homologues.

| **Phage region** | **φCD508 gene product** | **Phage/**  **PTLP** | **Protein** | | **PDB** | **Z-score** | **RMSD** | **Sequence Id %** |
| --- | --- | --- | --- | --- | --- | --- | --- | --- |
| **Head** | **gp45 Portal protein** | SPP1 | Portal protein | | 2jes | 18.5 | 4.4 | 14 |
|  |  | G20C | Portal protein | | 4zjn | 18.4 | 4.6 | 12 |
|  |  | RcGTA | Rcc01684 | | 6toa | 17.2 | 3.9 | 10 |
|  |  | T7 | Portal protein | | 7bou | 15.9 | 5.1 | 10 |
|  |  | Pam1 | Pam1 portal protein | | 7eep | 15.8 | 5.8 | 9 |
|  |  | T4 | Portal protein gp20 | | 3ja7 | 15.3 | 5 | 8 |
|  |  | P22 | Portal protein | | 5gai | 11.2 | 3.8 | 7 |
|  | **gp49 Major capsid protein** | HK97 | Major capsid protein | | 1ohg | 14.8 | 4.2 | 10 |
|  |  | RcGTA | Rcc01687 | | 6tsw | 13.2 | 3.7 | 9 |
|  |  | YSD1 | YSD1_17 | | 6xgq | 13 - 49 -.1 | 4.3 | 4 |
|  |  | T7 | Major capsid protein 10A | | 3j7w | 12.8 | 4.2 | 12 |
|  |  | T4 | Major capsid protein | | 5vf3 | 12.8 | 3.2 | 8 |
|  |  | Pam1 | Major capsid proteins | | 7eel | 12.4 | 4.9 | 9 |
|  | **gp48 Capsid decoration protein** | TW1 | Capsid Stabilizing Protein | | 5wk1 | 6.4 | 3.1 | 9 |
|  |  | XM1 | Minor capsid protein | | 7kmx | 6.3 | 3.2 | 15 |
|  |  | phi29 | gp8.5 Capsid fiber protein | | 6qyy | 5.7 | 3.3 | 12 |
|  |  | YSD1 | YSD1_16 | | 6xgq | 5.1 | 14.3 | 8 |
| **Neck** | **gp53 Tail terminator** | RcGTA | RCC01690 | | 6te9 | 13.2 | 2.8 | 14 |
|  |  | Lambda | Minor tail protein U | | 3fz2 | 12 | 3.3 | 10 |
|  |  | Pyocin | PA0615 | | 6u5j | 11.6 | 9.7 | 11 |
|  |  | AFP | Afp16 | | 6rap | 8.5 | 6.9 | 9 |
|  |  | T4 | Gp15 | | 3j2m | 8.4 | 6.4 | 8 |
|  |  | SPP1 | Gp17 | | 2lfp | 7.8 | 3.5 | 13 |
|  | **gp50 head-to-tail adaptor** | no known structural homologues | |  | | | | |
|  | **gp51 Neck valve protein** | RcGTA | RCC01688 | | 6te9 | 4 | 3.2 | 7 |
|  |  | PBSX | PBSX protein XkdH | | 3f3b | 2.6 | 4.2 | 5 |
| **Tail** | **gp55 Tail sheath** | Pyocin | sheath | | 3j9q | 30.3 | 3.3 | 12 |
|  |  | Diffocin | Putative phage XkdK-like protein | | 6gkw | 29.6 | 2.5 | 22 |
|  |  | AFP | AFP3 | | 6rao | 26 | 3.9 | 9 |
|  |  | T4 | Tail sheath protein Gp18 | | 3j2m | 23.8 | 3.9 | 16 |
|  |  | phi812K1-420 | tail sheath protein | | 5li2 | 20.3 | 3.7 | 13 |
|  |  | T6SS | type VI secretion protein | | 5mxn | 18.2 | 9.2 | 9 |
|  | **gp56 Tail tube** | AFP | AFP1 | | 6rap | 12 | 2.7 | 11 |
|  |  | SPP1 | Tail tube protein gp17.1 | | 6yeg | 9.9 | 2.5 | 12 |
|  |  | T4 | Tail tube protein gp19 | | 5w5f | 9.8 | 2.6 | 11 |
|  |  | Pyocin | FIIR2 protein | | 5w5e | 9.5 | 2.5 | 14 |
|  |  | YSD1 | YSD1_22 major tail protein | | 6xgr | 9.2 | 3 | 6 |
|  |  | T5 | Tail tube protein | | 5ngj | 8.4 | 2.3 | 13 |
| **Baseplate** | **gp65 Triplex 1a/b** | PVC | Pvc11 | | 6j0n |  |  |  |
|  |  | Pyocin | PA0618 | | 6u5b |  |  |  |
|  |  | AFP | Afp11 | | 6rao | 12.6 | 16.2 | 9 |
|  |  | T4 | gp6 | | 5hx2 | 8.2 | 12 | 16 |
|  |  | XM1 | gp16 | | 7kh1 |  |  |  |
|  | **gp61 Tail tube initiator** | AFP | Afp7 | | 6rao | 10.6 | 10.2 | 13 |
|  |  | P2 | ORF15 | | 2wzp | 9 | 6.2 | 10 |
|  |  | SPP1 | gp 19.1 | | 2x8k | 7.9 | 6.7 | 7 |
|  |  | RcGTA |  | | 6toa | 7.6 | 3.7 | 9 |
|  |  | SPP1 | gp17.1 | | 6yeg | 7.1 | 3.1 | 11 |
|  |  | YSD1 | YSD1_22 | | 6xgr | 6.9 | 3.2 | 11 |
|  | **gp64 Sheath initiator** | AFP | Afp9 | | 6rao | 9.7 | 2.5 | 21 |
|  |  | T4 | gp25 | | 5iw9 | 7.7 | 5.6 | 11 |
|  |  | T6SS | TssE | | 6gj1 | 6.8 | 3.2 | 11 |
|  |  | RcGTA | Rcc01690 | | 6te9 | 4.4 | 3.4 | 5 |
|  | **gp66 Triplex 2** | T4 | gp6 | | 5hx2 | 6.5 | 7.1 | 8 |
|  |  | AFP | Afp11 | | 6rao | 5.9 | 7.6 | 8 |
| **Needle** | **gp63 Needle tip** | PVC | Pvc8 | | 6j0m | 5.0 | 5.7 | 10 |
|  |  | Phage SN | puncturing protein gp41 | | 4ru3 | 4.6 | 4.1 | 14 |
|  |  | P2 | Baseplate assembly protein V | | 3qr8 | 4.5 | 4.1 | 12 |
|  |  | Pyocin | PA0616 | | 4s37 | 4.4 | 4.3 | 10 |
|  |  | T6SS | VgrG1 | | 4uhv | 3.5 | 6.5 | 13 |
|  | **gp62 Needle** | Pyocin | hub | | 6u5h | 19.6 | 3.0 | 10 |
|  |  | PVC | Pvc8 | | 6j0m | 19.0 | 3.3 | 15 |
|  |  | T6SS | VgrG1 | | 4uhv | 18.7 | 3.2 | 11 |
|  |  | T4 | Baseplate structural protein Gp27 | | 2z6b | 18.4 | 3.2 | 10 |
|  |  | P2 |  | | 2wzp | 10.7 | 4.1 | 11 |
|  |  | RcGTA |  | | 6teh | 9.2 | 3.5 | 8 |
